# A Point in the Treatment of Mammary Abscesses

**Published:** 1848-07

**Authors:** 


*A Point in the treatment of Mammary Abscesses.*—"The following is a case of no very unusual occurrence:—A mother loses her infant, and neglects to pay that attention to herself which her case requires. No attempts being made to relieve the distended breast of its secretion, the gland becomes hard and painful, and at length abscesses form in different situations. If the treatment is confined to the evacuation of matter by puncture, and to the application of fomentations and poultices, but little good is done. The former abscesses continue to discharge, fresh ones are constanty forming, and the woman at length sinks into a state of hectic. Means should be used to stop the secretion of milk, which has been going on all this time, and perpetuating the mischief. We have no better means of effecting this than by the ad ministration of a hydragogue purgative. I am usually in the habit of prescribing the sulphate of magnesia in the compound infusion of roses, to which, when there is much hectic and debility, I add some quinine and dilute sulphuric acid. The effect of this treatment is sometimes almost magical. I have known a woman, who for months had been suffering from a succession of mammary abscesses, begin to get well from the moment the salts produced its liquid evacuations from the bowels, ; the secretion of milk ceased, and the purulent discharge diminished, a more adhesive inflammation being established in the place of these two actions."—*Provincial Journal.*

